# Editorial: Traditional, complementary and integrative medicine – opportunities for managing and treating neurodegenerative diseases and ischaemic stroke

**DOI:** 10.3389/fphar.2025.1749624

**Published:** 2025-12-15

**Authors:** Huazheng Liang, Chunguang Li, Bin Yu

**Affiliations:** 1 Translational Research Institute of Brain and Brain-Like Intelligence, Shanghai Fourth People’s Hospital, School of Medicine, Tongji University, Shanghai, China; 2 Shanghai Key Laboratory of Anesthesiology and Brain Functional Modulation, Shanghai, China; 3 Clinical Research Center for Anesthesiology and Perioperative Medicine, Shanghai Fourth People’s Hospital, School of Medicine, Tongji University, Shanghai, China; 4 Suzhou Industrial Park Monash Research Institute of Science and Technology, Suzhou, Jiangsu, China; 5 Southeast University-Monash University Joint Graduate School, Suzhou, Jiangsu, China; 6 Monash University- Southeast University Joint Research Institute, Suzhou, Jiangsu, China; 7 Australian Regenerative Medicine Institute, Clayton, VIC, Australia; 8 NICM Health Research Institute, Western Sydney University, Westmead, NSW, Australia; 9 Jiangsu Key Laboratory for Pharmacology and Safety Evaluation of Chinese Materia Medica, Nanjing University of Chinese Medicine, Nanjing, Jiangsu, China

**Keywords:** clinical trial, gut microbiota, ischemic stroke, natural products, neurodegenerative diseases, pharmacology, therapeutic targets

Ischemic stroke and Alzheimer’s disease (AD) are leading neurological causes of death and disability (Avan and Hachinski, 2023; An et al., 2025), yet both face profound research and therapeutic challenges. Stroke treatment is constrained by a narrow therapeutic window, thrombolysis and endovascular therapy must be administered within hours (Ho and Powers, 2025), limiting accessibility, especially in rural or underserved regions. Post-stroke care is complicated by heterogeneous patient presentations, variable reperfusion outcomes, and a lack of neuroprotective agents that translate from animal models to humans. Chronic inflammation, blood-brain barrier disruption, and comorbidities like diabetes or atrial fibrillation further obscure treatment efficacy and recovery trajectories (Pandian et al., 2023; Zeng et al., 2025).

Alzheimer’s disease research grapples with its long preclinical phase, during which pathology silently accumulates decades before symptoms emerge. This complicates early diagnosis and intervention (Marvi et al., 2025). The field has been dominated by the amyloid cascade hypothesis, yet anti-amyloid therapies have shown only modest cognitive benefits, raising questions about causality and timing (Reiss et al., 2021). Tau pathology, neuroinflammation, vascular dysfunction, and synaptic loss are increasingly recognized as multifactorial drivers (Rajendran and Krishnan, 2024; Cai et al., 2025) but integrating these into cohesive treatment strategies remains elusive. Biomarker development is advancing, with CSF and PET imaging improving diagnostic accuracy, but high cost, limited availability, and invasiveness hinder widespread use (Krishnamurthy et al., 2025).

This research topic comprises 16 published papers tackling the abovementioned challenges. Apart from conventional secondary prevention medications, there are few drugs available to treat ischemic stroke patients. In a study by Wu et al., Shenmai Injection given to ischemic stroke patients for 10 days after rt-PA thrombolysis significantly lowered 30-day mRS scores compared with placebo, indicating better functional recovery in ischemic stroke patients without added adverse events. In H_2_O_2_-stressed PC12 cell culture model, Shenmai Injection significantly enhanced cell viability, reduced apoptosis, diminished the levels of reactive oxygen species and malondialdehyde. In the meantime, it increased the level of ATP and mitochondrial potential. These protective effects are abolished by AMPKα1 knockdown, indicating that these protective effects are partially mediated by AMPKα1 (Wu et al.).

In a real-world, multi-center retrospective study, Pan et al. found that Danhong Injection as an adjunct therapy significantly improved neurological outcomes in patients with acute ischemic stroke, evidenced by lower National Institutes of Health Stroke Scale (NIHSS) at discharge and higher proportions of patients achieving NIHSS ≤4 and ≤1 compared with the control group. The drug group also showed better functional recovery, with more patients achieving modified Rankin Score (mRS) ≤1. There was no significant difference in in-hospital complications (Pan et al.). These findings suggest Danhong Injection may enhance short-term recovery in acute ischemic stroke patients and further randomized trials are needed.

In a systematic review and meta-analysis by Wu et al., results from 51 RCTs (9,577 patients) found that Tongxinluo significantly improves stroke outcomes including enhancing the functional recovery through decreasing the NIHSS, improving lipid profiles, and inflammation markers without increasing adverse events. Benefits were greater in patients ≤65 years and with ≤4 weeks treatment. However, heterogeneity and publication bias limits the fidelity (Wu et al.).

Xu et al. summarized the progress on *Bovis Calculus’s* mechanism and application in ischemic stroke. This traditional medicine shows strong potential in treating ischemic stroke by mitigating oxidative stress, inflammation, and apoptosis while promoting angiogenesis and neurogenesis. Its active components, including bile acids and taurine, act through multiple pathways to protect brain tissue. Clinical studies support its safety and efficacy, especially in improving neurological function and recovery (Xu et al., 2025).

A review by Sowmiya et al. demonstrates that Traditional, Complementary and Integrative Medicine (TCIM) offers multi-targeted, clinically relevant support for ischemic-stroke patients. Evidence from controlled trials and mechanistic studies indicates that standardized herbal formulas, such as *Salvia miltiorrhiza Bunge [*Lamiaceae*; Salviae miltiorrhizae radix et rhizoma], Ginkgo biloba L. [*Ginkgoaceae*; Ginkgo folium], and Panax ginseng C.A. Mey. [*Araliaceae*; Ginseng radix]*, acupuncture, yoga, polyphenol-rich diets, aromatherapy and music therapy can significantly reduce oxidative stress, neuroinflammation, mitochondrial dysfunction, ferroptosis and autophagy, while enhancing antioxidant defenses, cerebral blood flow, neurogenesis and angiogenesis. These interventions improve neurological scores, functional independence and quality of life when used adjunctively with thrombolysis or rehabilitation. However, benefits are constrained by heterogeneous formulations, small sample sizes, short follow-ups and scarce herb-drug interaction data. The review, therefore, calls for large, multi-center, double-blind randomized trials with defined phytochemical profiles, biomarker endpoints and systems-biology analyses to validate efficacy, optimize dosing and facilitate safe, evidence-based integration of TCIM into mainstream stroke management protocols worldwide (Sowmiya et al.).

Another review by Niu et al. further highlighted the therapeutic potential of natural plant compounds in modulating neuroinflammation following ischemic stroke. It systematically summarizes how polyphenols, flavonoids, saponins, diterpenoids, and alkaloids exert anti-inflammatory effects by targeting key signalling pathways, such as NF-κB, NLRP3, TLR4, and JAK/STAT. These compounds inhibit microglial activation, reduce pro-inflammatory cytokines, and suppress immune cell infiltration, thereby attenuating post-stroke brain injury. Despite promising preclinical results, challenges like poor bioavailability, limited BBB penetration, unclear molecular targets, and translational gaps remain. This review emphasizes the need for advanced delivery systems, mechanistic clarification, and rigorous clinical trials to advance these natural products as viable neuroprotective therapies for ischemic stroke (Niu et al.).

A review by Zheng et al. identified ferroptosis, an iron-dependent form of regulated cell death, as a key therapeutic target in both ischemic and hemorrhagic stroke. It systematically summarized mechanisms driving ferroptosis, including iron overload, lipid peroxidation, and glutathione depletion. Authors analyzed 55 preclinical studies and highlighted that natural products, such as ginkgolide B, quercetin, berberine, and salvianolic acid A, effectively inhibited ferroptosis *via* the Nrf2/HO-1, GPX4, and SLC7A11 pathways. Despite promising animal data, clinical translation remains limited due to issues like poor bioavailability, unclear targets, and lack of standardized formulations. This review emphasizes the need for pharmacokinetic profiling, target validation, and robust clinical trials to advance these natural compounds into stroke therapeutics (Zheng et al.).

Alzheimer’s disease (AD) remains a challenging medical condition with no effective treatments. However, traditional medicine offers some empirical recipes to halt the progression of this condition. A study by Liu et al. demonstrated that Kai-Xin-San (KXS), a traditional Chinese herbal formula, improved cognitive functions in SAMP8 mice, a model of mild cognitive impairment, by reducing amyloid-beta deposition, suppressing neuroinflammation, and inhibiting pyroptosis. These protective effects are mediated through modulating the NLRP3/Caspase-1 signalling pathway (Liu et al., 2025). These findings suggest that KXS is a promising multi-target therapeutic strategy for early-stage cognitive decline.

In another study by Lin et al., NMN was reported to rescue D-galactose-induced aging in mice by restoring NAD^+^, activating Sirt1/AMPK/PGC-1α, alleviating oxidative stress, neuroinflammation and apoptosis, while enhancing neurotransmission and rebuilding the intestinal barrier. All these benefits vanished when a Sirt1 inhibitor Ex527 was applied, proving that these benefits are mediated through Sirt1 (Lin et al., 2025).

In an APP/PS1 mouse model of AD, Bai et al. reported that Icariside II, the most potent *Epimedii Folium* metabolite, attenuated cognitive deficits of these mice through restoring hippocampal neurogenesis. This benefit required intact mitochondria, as it normalized fusion/fission proteins, ATP and ROS, yet vanished when rotenone disrupted mitochondrial functions. Other metabolites of *Epimedii Folium* including Icarlin and Icaritin also showed similar effects but they were weaker compared to Icariside II (Bai et al., 2025).

In a scopolamine-induced rat model of AD, Rastinpour et al. showed that Astaxanthin significantly mitigated cognitive deficits by enhancing antioxidant defenses (catalase, glutathione), reducing oxidative stress (nitrite), and modulating inflammatory markers (MMP-2↑, MMP-9↓). It also regulated key transcription factors like upregulating Nrf2 and downregulating NF-κB while preserving hippocampal neurons. Notably, these neuroprotective effects were partially reversed by opioid (naloxone) and GABA-A (flumazenil) receptor antagonists, indicating that astaxanthin’s benefits involve modulation of these neurotransmitter systems (Rastinpour et al.).

In a similar model of AD, Yang et al. demonstrated that Xinshubao tablet (XSB), a traditional Chinese medicine, significantly improved cognitive functions. Using a multi-omics approach, the study revealed that XSB mitigated neuroinflammation, oxidative stress, and cholinergic dysfunction while enhancing synaptic integrity. XSB modulated gut microbiota by increasing beneficial bacteria (e.g., *Enterococcus*, *Actinobacteriota*) and reducing harmful species (*Helicobacter rodentium*). It also restored metabolic balance by regulating pathways, such as glycerophospholipid and unsaturated fatty acid metabolism. Fecal microbiota transplantation from XSB-treated mice replicated these cognitive and intestinal barrier benefits, confirming that the gut-brain axis is a key pathway for mediating the protective effect. These findings highlight XSB’s potential as a multifactorial therapeutic strategy for AD (Yang et al.).

In an aluminum chloride-induced rat AD model, Zarneshan et al. reported that polydatin dose-dependently reversed memory deficits and anxiety-like behaviors, performing comparably to donepezil. Polydatin elevated antioxidant defenses (glutathione, catalase), reduced oxidative nitrite, shifted matrix metalloproteinase balance (downregulated pro-inflammatory MMP-9, upregulated protective MMP-2), and preserved hippocampal CA2, CA4 and dentate gyrus neurons. These antioxidant, anti-inflammatory and neuroprotective mechanisms underlie polydatin’s therapeutic potential against AD (Zarneshan et al., 2025).

An LC-MS/MS study by Hu et al. revealed that oral ginsenoside Rh3 (GRh_3_) exhibited sustained-release pharmacokinetics in rats: delayed Tmax (8 h), long half-life (14.7 h), low clearance (13 L/h/kg) and large volume of distribution (280 L/kg). After being administered at 100 mg/kg, the compound accumulated most in intestine > stomach > liver, but notably penetrated brain tissue, reaching 520 ng/g in the hippocampus, which is contradicting the rule that only <500 Da molecules can cross the blood brain barrier. This brain enrichment, coupled with prolonged systemic exposure, supports GRh3’s potential for chronic neuroprotective therapies and warrants investigation of transporter-mediated BBB mechanisms (Hu et al.).

In another study, Sneha et al. reported the therapeutic potential of Traditional, Complementary, and Integrative Medicine (TCIM) as an adjunct to conventional treatments for AD. Unlike standard pharmaceuticals that offer only symptomatic relief, TCIM employs a holistic, multi-targeted approach, addressing key pathological mechanisms, such as amyloid-beta aggregation, tau hyperphosphorylation, oxidative stress, neuroinflammation, and mitochondrial dysfunction. Promising herbal agents including *Withania somnifera (L.) Dunal*, *Curcuma longa L*, *Bacopa monnieri (L.) Wettst*, *Centella asiatica (L.) Urb*, and *G. biloba L* demonstrate neuroprotective, anti-inflammatory, and antioxidant properties in preclinical and limited clinical studies. Mind-body practices like meditation also contribute to the therapeutic effect by reducing stress, a known modifiable risk factor. However, the review emphasized that despite encouraging results, most TCIM studies suffer from methodological limitations, including small sample sizes, lack of standardization, poor bioavailability, and insufficient randomized controlled trials. To advance TCIM’s role in AD management, future research must prioritize rigorous, large-scale, multi-center randomized controlled trials, pharmacokinetic profiling, herb-drug interaction studies, and personalized, integrative therapeutic strategies combining traditional and modern medicine (Sneha et al.).

Parkinson’s disease (PD) is the second most common neurodegenerative disease. No cure is available for this condition. In the review by Zhou and Pan, the therapeutic potential of polysaccharides derived from Traditional Chinese Medicine in treating PD through multiple mechanisms has been summarized. These polysaccharides, extracted from sources like *Astragalus mongholicus Bunge [*Fabaceae*, Gastrodia elata Blume [*Orchidaceae*]*, *Lycium barbarum L. [*Solanaceae*]*, and *Cistanche deserticola Ma [*Orobanchaceae*]*, exhibit neuroprotective effects by inhibiting apoptosis, reducing oxidative stress, enhancing mitochondrial function, promoting autophagy, and alleviating neuroinflammation. They also modulate key signalling pathways, such as PI3K/AKT/mTOR and AMPK/mTOR, and may influence the gut-brain axis by restoring microbiota balance. Preclinical studies showed improvements in motor and non-motor symptoms in PD models, with some evidence of dopaminergic neuron protection and α-synuclein aggregation inhibition. Despite promising results, clinical translation is limited by lack of standardized formulations, bioavailability issues, and absence of large-scale human trials. Future research should focus on clinical validation, mechanism elucidation, and formulation optimization to advance these polysaccharides as viable PD therapeutics (Zhou and Pan).

Taking these studies together, this Research Topic has provided pharmaceutical and clinical evidence on naturally derived products in managing ischemic stroke and neurodegenerative diseases like AD and PD. They take therapeutic effects through multiple mechanisms. However, robust clinical evidence is still needed to overcome limitations of current studies, such as small sample sizes, patient heterogeneity, and poor translational potential. Nevertheless, these natural products might become drug candidates against ischemic stroke and neurodegenerative diseases and open a new era of drug development.
